# Clinical Verification of A Clinical Decision Support System for Ventilator Weaning

**DOI:** 10.1186/1475-925X-12-S1-S4

**Published:** 2013-12-09

**Authors:** Jiin-Chyr Hsu, Yung-Fu Chen, Wei-Sheng Chung, Tan-Hsu Tan, Tainsong Chen, John Y Chiang

**Affiliations:** 1 Institute of Biomedical Engineering, National Cheng Kung University, Tainan, Taiwan; 2 Department of Internal Medicine, Taoyuan General Hospital, Ministry of Health and Welfare, Taoyuan, Taiwan; 3 Department of Healthcare Administration, Central Taiwan University of Science and Technology, Taichung, Taiwan; 4 Department of Health Services Administration, China Medical University, Taichung, Taiwan; 5 Department of Internal Medicine, Taichung Hospital, Ministry of Health and Welfare, Taichung, Taiwan; 6 Department of Electrical Engineering, National Taipei University of Technology, Taipei, Taiwan; 7 Department of Computer Science and Engineering, National Sun Yat-sen University, Kaohsiung, Taiwan

**Keywords:** Ventilator weaning, Clinical Decision Support System, Healthcare cost

## Abstract

**Background:**

Weaning is typically regarded as a process of discontinuing mechanical ventilation in the daily practice of an intensive care unit (ICU). Among the ICU patients, 39%-40% need mechanical ventilator for sustaining their lives. The predictive rate of successful weaning achieved only 35-60% for decisions made by physicians. Clinical decision support systems (CDSSs) are promising in enhancing diagnostic performance and improve healthcare quality in clinical setting. To our knowledge, a prospective study has never been conducted to verify the effectiveness of the CDSS in ventilator weaning before. In this study, the CDSS capable of predicting weaning outcome and reducing duration of ventilator support for patients has been verified.

**Methods:**

A total of 380 patients admitted to the respiratory care center of the hospital were randomly assigned to either control or study group. In the control group, patients were weaned with traditional weaning method, while in the study group, patients were weaned with CDSS monitored by physicians. After excluding the patients who transferred to other hospitals, refused further treatments, or expired the admission period, data of 168 and 144 patients in the study and control groups, respectively, were used for analysis.

**Results:**

The results show that a sensitivity of 87.7% has been achieved, which is significantly higher (*p*<0.01) than the weaning determined by physicians (sensitivity: 61.4%). Furthermore, the days using mechanical ventilator for the study group (38.41 ± 3.35) is significantly (*p*<0.001) shorter than the control group (43.69 ± 14.89), with a decrease of 5.2 days in average, resulting in a saving of healthcare cost of NT$45,000 (US$1,500) per patient in the current Taiwanese National Health Insurance setting.

**Conclusions:**

The CDSS is demonstrated to be effective in identifying the earliest time of ventilator weaning for patients to resume and sustain spontaneous breathing, thereby avoiding unnecessary prolonged ventilator use and decreasing healthcare cost.

## Introduction

Weaning is typically regarded as a process of discontinuing mechanical ventilation in the daily practice of an intensive care unit (ICU). It was reported that, 39%-40% need mechanical ventilator for sustaining their lives among the ICU patients [1]. For the ICU patients with ventilator, 90% of them can be weaned from the ventilator within a few days [2], while other patients need longer ventilator support [3,4]. In order to reduce the likelihood of known nosocomial complications and costs, ventilator support should be withdrawn promptly when no longer necessary [5,6]. Risks of subglottic injury, respiratory infections, and chronic lung disease increases because of prolonged use of ventilator [7]. To discontinue mechanical ventilation and remove the artificial airway as soon as possible can reduce the risk of ventilator-induced lung injury (VILI), nosocomial pneumonia, airway trauma from the endotracheal tube, and unnecessary sedation. On the other hand, premature ventilator-discontinuation or extubation can cause respiratory muscle fatigue, gas exchange failure, loss of airway protection, and an increase of patient mortality [8-10]. Hence, to begin weaning process at the right time is very important, but difficult in the clinical practice.

It was reported that the rate of successful weaning achieved only 35-60% if the decisions were made by physicians [11-13]. In general, physicians' judgments are prone to be unreliable; therefore, decreasing the dependence on their individual knowledge, experience, and skill is promising in elevating the predictive rate if objective measurements and effective weaning variables can be administrated. In previous studies, several physiological variables including rapid shallow breathing index measures by frequency-to-tidal volume ratio (f/VT), maximal inspiration pressure (PImax), vital capacity (VC), minute ventilation (VE), pH and pCO2 values of stomach mucosa, arterial blood gas level, fraction of inspired oxygen, alveolar-arterial oxygen pressure difference (A-a gradient), blood urine nitrogen (BUN) level, serum creatinine level, and serum albumin level, have been reported to be useful for weaning prediction [14-20]. However, there is still no agreement being made so far to determine which variables should be monitored [12,13]. Furthermore, previous studies focused only on the physiological variables, other factors including diseases, such as pulmonary, cardiac, respiratory, and brain vessel diseases, as well as therapeutic progression indexes, i.e. acute physiology and chronic health evaluation II (APACHE II) and coma scales, were seldom considered. It was reported that a predictor designed with variables obtained from a single device tends to incur systematic errors [21]. Adoption of multiple variables acquired from various modalities is effective in eliminating systematic errors.

Clinical decision support systems (CDSSs) are promising in providing useful information and expert knowledge to enhance diagnostic performance and improve healthcare quality in clinical setting. It was reported that among the 97 proposed CDSS applications, including 10 diagnostic systems, 21 reminder systems, 37 disease management systems, and 29 drug-dosing or prescribing systems, 64% of the CDSSs demonstrated improved outcomes in medical practice [22]. CDSSs have been applied in the diagnoses of lower back pain [23], otological disease [24], cardiovascular disease [25], and cancer using endoscopic images [26]; management and care of chronic heart failure [27] and chronic kidney failure [28]; and management of operational risk in hemodialysis [29]. Recently, CDSSs were also applied to care patients who needed mechanical ventilation to sustain their lives [30-32]. It was useful for nurses to increase their adherence to guidelines and to improve the positioning of patients who received mechanical ventilation by implementing a CDSS as an electronic reminder [30]. Variables including pulmonary and gastrointestine diagnoses, body mass index, and tube-feeding were reported to be important for determining head-of-bed position for patients. It was also reported that a significant effect was found by adopting the CDSS to improve a guideline recommending the administration of lower tidal volume for ICU patients receiving mechanical ventilation longer than 24 hours [31]. It is effective in preventing patients with acute lung injury from ventilator-associated lung injury.

The objective of the study is to validate effectiveness of the designed CDSS in the clinical setting. Before prospective evaluation, an evaluation of the CDSS must be conducted retrospectively [33]. In our previous report, the CDSS designed with support vector machine (SVM) embedding a kernel of radial basis function (RBF) was trained and cross-validated using retrospective data containing 11 variables selected with recursive feature elimination (RFE) strategy. It was demonstrated to have a predictive rate as high as 92.73% in ventilation weaning [34]. To the best of our knowledge, a prospective study has never been conducted to verify the effectiveness of the CDSS in ventilator weaning. In this study, the following hypotheses are examined: (1) the CDSS is able to predict the weaning outcome with great sensitivity and (2) is able to reduce the duration of ventilator support for patients, resulting in a decrease of healthcare cost.

## Materials and methods

### Patients and setting

A prospective, randomized, and controlled trial was designed to verify if the CDSS designed for ventilator weaning is more effective than the traditional weaning determined only by physicians to assist physicians to wean critically ill patients from the mechanical ventilators. The study was conducted in a 16-bed respiratory care center (RCC) of a regional teaching hospital in the central region of Taiwan from Jan 2008 to July 2009. The trial was registered at Institutional Review Board of Taichung Hospital, Ministry of Health and Welfare, Executive Yuan, Taichung, Taiwan, with No. B980003.

**Figure 1:**
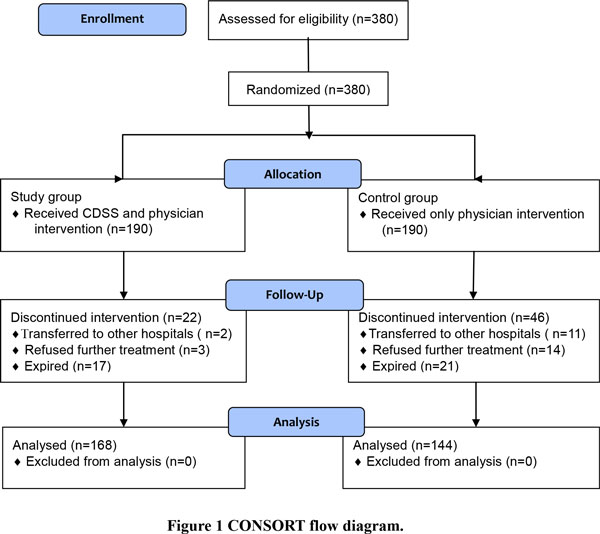
**CONSORT flow diagram**.

A total of 380 patients were admitted to the RCC of the hospital. They were randomly assigned, according to a randomized list generated by a computerized random generator, to one of the two groups: (1) control group- patients weaned with traditional protocol determined only by physicians and (2) study group- patients weaned using CDSS monitored by physicians. In the study group, if the CDSS predicted that a patient could be successfully weaned from the ventilator and confirmed by the attending physician, the weaning process was initiated. In the control group, a patient judged by the attending physician to be ready for ventilator weaning was eligible to initiate the weaning process. Figure 1 shows the Consort flow diagram.

**Figure 2:**
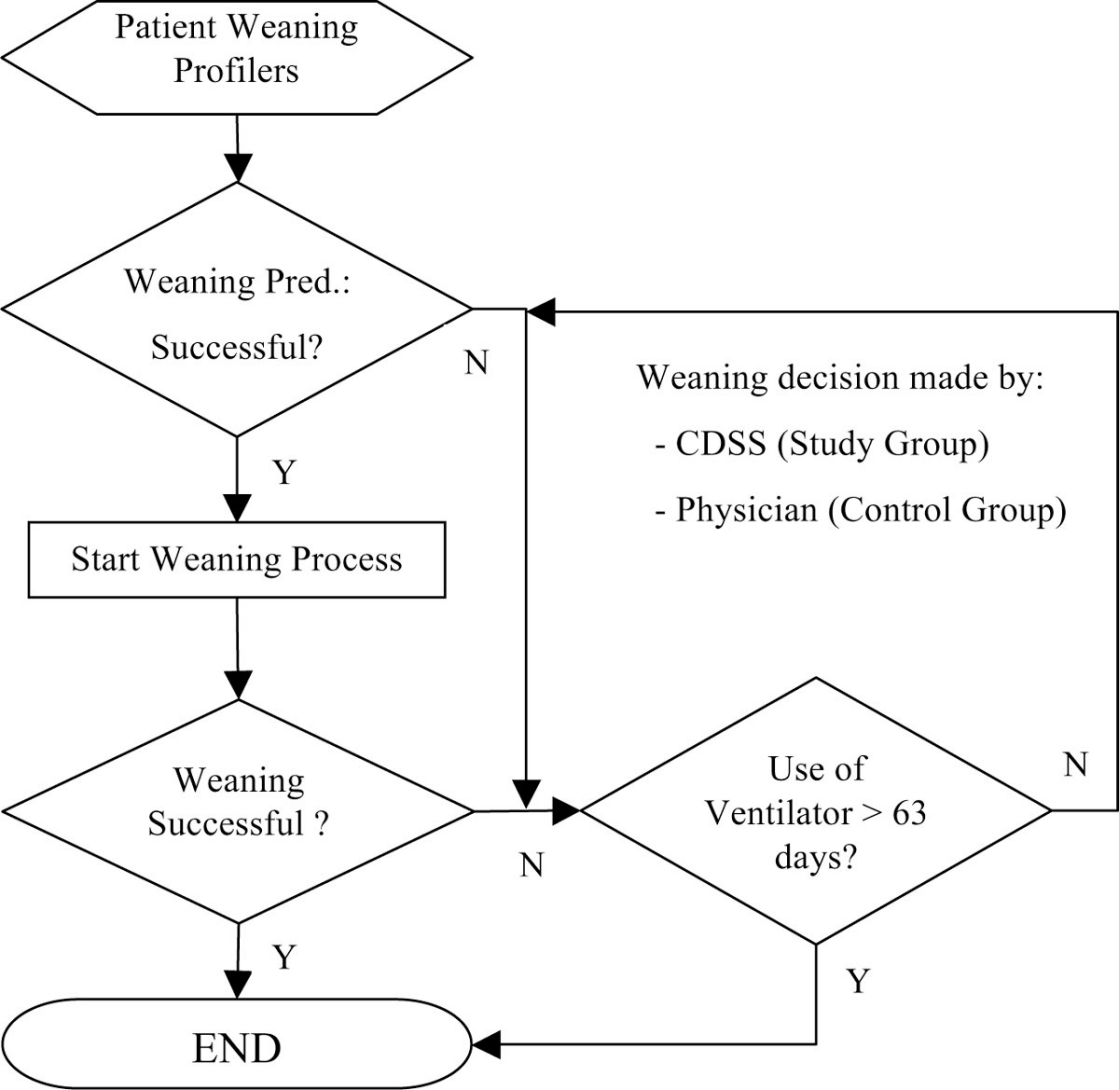
**Experimental procedure**.

The experimental procedure is shown in Figure 2. As illustrated in this figure, patients who failed in ventilation weaning with a period of using ventilator for less than 63 days were reintubated and waited for another spontaneous breathing test (SBT).

**Figure 3:**
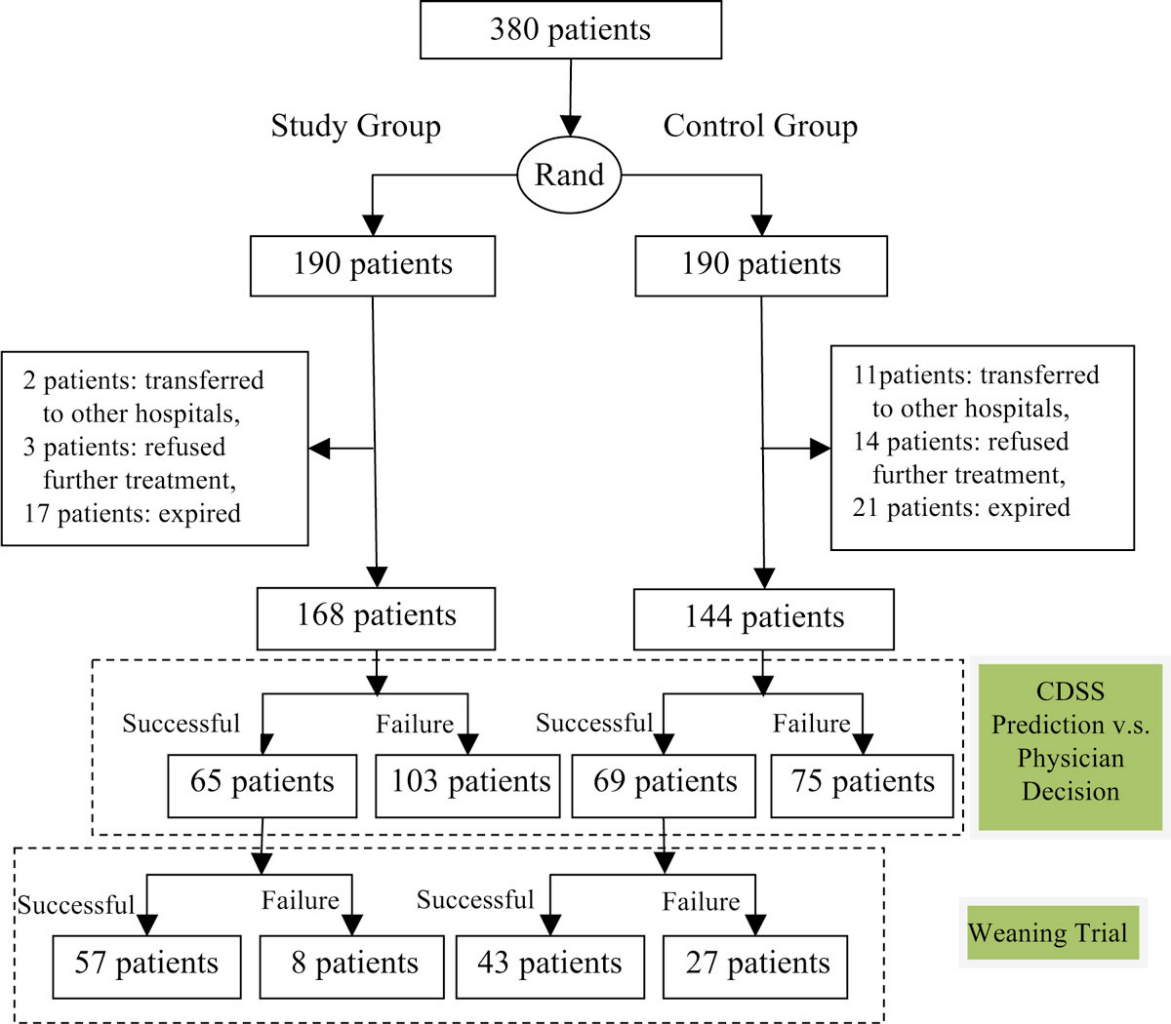
**Experimental paradigm**.

As shown in Figure 3, the recruited patients were randomly assigned to either one of the two groups, each consisting of 190 patients. Because of transferring to other hospitals, refusing to receive further treatments, or expiring after having used ventilator for more than 63 days, 22 patients in the study group and 46 in the control group, respectively, were terminated, resulting in 168 and 144 patients in two individual groups for further study.

### Weaning protocol

Patients eligible for weaning, in a semi-recumbent position, were submitted to 60 minute of SBT. Endotracheal suctioning was performed before SBT. Patients were allowed to breathe through the ventilatory circuit by using flow triggering (2 L/min) with automatic tube compensation (ATC) of 100% and positive end expiratory pressure (PEEP) of 5 cmH_2_O. The inspired oxygen (FiO_2_) was set to the same value during mechanical ventilation.

The tolerance to SBT was considered poor in the presence of at least one of the follow criteria: a decrease in oxygen saturation to <90% while requiring FiO_2_>0.5; evidence of respiratory distress; sustained increase in HR (>140 bpm or >20% of the baseline); systolic blood pressure (SBP) >200 mmHg or <90 mmHg, or SBP change >20% of the baseline; uncoordinated thoraco-abdominal movement; and agitated or depressed mental status.

If the patients could clinically well tolerate the 60-minute SBT, they were extubated immediately or would use T-piece if undergone tracheotomy. Patients who did not tolerate the SBT were reconnected to the ventilator. The decisions to reconnect the patients were made by the attending physicians.

Patients were classified as either weaning successfully (WS) or weaning failingly (WF). The WS was defined as the ability to maintain unassisted breathing spontaneously for >72 hours after discontinuing from a mechanical ventilator [35]. On the other hand, the WF patients were those who died after discounting from a mechanical ventilator, reintubated after extubation, or required the support of a mechanical ventilator within 72 hours. The successful weaning rate was defined as the number of patients who had been successfully weaned to the number of patients who was admitted to the ICU and used ventilator for sustaining their lives.

### Design of clinical decision support system

In this study, a CDSS was applied to prospectively predict if a patient can be successfully weaned from mechanical ventilators. A total of 348 data containing 27 variables, including demographic information, physiology and disease factors, and care and treatment factors, were used for CDSS design. Table 1 shows the descriptive and inference statistics of the 27 individual variables of collected data. For inferential studies, continuous variables were analyzed with Student's *t*-test, while categorical variables with Pearson *χ*^2 ^test. As shown in this table, 15 variables (7 continuous variables and 8 discrete variables) are demonstrated to have significant difference (*p*<0.05) between successful and failed weaning groups based on single-variable analysis. The CDSS was designed based on the methods reported in our previous study [34], as summarized below:

**Table 1:** Statistic analyses of 27 recorded variables of collected data (N = 348)

Variables	Ventilator Weaning		Significance (*p*-value)
		
	Successful (n = 159)		Failed (n = 189)
**Demographic Data**			
Gender (male/female)	90/69	110/79	0.828
Age***	72.67 ± 14.95	78.04 ± 12.22	<0.001

**Physiology and Disease Factors**			
APACHE II score at hospitalization*	17.21 ± 4.94	18.62 ± 5.83	0.017
Coma Scale at hospitalization	9.54 ± 3.37	9.34 ± 3.93	0.611
Albumin (mg/dl)	2.78 ± 0.48	2.86 ± 2.77	0.725
Blood urea nitrogen (BUN) (mg/dl) ***	25.95 ± 17.45	39.26 ± 29.77	<0.001
Creatinine (mg/dl)	1.25 ± 0.92	3.97 ± 20.51	0.095
Hemoglobin (g/dl)	10.92 ± 1.61	10.46 ± 5.52	0.311
Pulmonary disease	92 (57.9%)	101 (53.4%)	0.449
Cardiac disease	26 (16.4%)	36 (19.0%)	0.575
Historical respiratory disease^‡^	32 (20.1%)	70 (37.0%)	0.001
Brain vessel disease	18 (11.3%)	14 (7.4%)	0.264
Other causes related to int. medicine^‡^	80 (50.3%)	129 (68.3%)	0.001
Acute respiratory distress syndrome	3 (1.9%)	0 (0%)	0.094
Multiple-organ failure^†^	0 (0%)	8 (4.2%)	0.009
Trauma	0 (0%)	4 (2.1%)	0.128
Brain Surgery^‡^	41 (25.8%)	19 (31.7%)	<0.001
Other kinds of surgeries^†^	8 (5.0%)	24 (12.7%)	0.015

**Care and Treatment Factors**			
Tracheotomy	75 (47.2%)	71 (37.6%)	0.081
Coma scale at weaning***	9.23 ± 2.94	7.37 ± 2.96	<0.001
RSBI at weaning***	91.59 ± 41.94	162.58 ± 79.62	<0.001
Length of ICU admission (day)*	16.72 ± 6.54	18.26 ± 6.35	0.026
Days using ventilator***	33.38 ± 12.01	41.24 ± 16.68	<0.001
Ventilator associated pneumonia^‡^	20 (12.6%)	77 (40.7%)	<0.001
Blood stream infection^‡^	0 (0%)	8(4.2%)	0.009
Urinary tract infection^‡^	19 (11.9%)	50 (26.5%)	0.001
Nosocomial infection	8 (5.0%)	10 (5.3%)	1.000

Note: *p<0.05, ** *p*<0.01, and ***p<0.001 with Student's *t*-test; ^†^*p*<0.05 and ^‡^*p*<0.01 with Pearson *χ*^2 ^test.

The predictive performance of feature selection strategies based on filter method and wrapper method were compared in our previous study [34]. The filter method is a preprocessing procedure selecting a subset of features based on statistic measures independent of the designed classifiers, while the wrapper method assesses individual subsets of features in a recursive way by considering their predictive efficiency to a given classifier. The filter approach is based on logistic regression analysis (LRA), which is a type of nonlinear regressions widely used for delineating the relationship between collection of several independent variables consisting of discrete and continuous types and a dependent discrete (dichotomous or multiple) variable. In contrast to single-variable analysis (Table 1), only 7 variables, i.e. blood urine nitrogen, brain surgery, coma scale at weaning, RSBI, days using ventilator, ventilator associated pneumonia, and urinary tract infection, were significant (*p*<0.05) for multivariate LRA. On the other hand, the recursive feature elimination (RFE) algorithm and SVM classifier were adopted for feature selection and sample classification, respectively, in the wrapper method. For a vector space with *n *features, RFE algorithm starts with all features and removes insignificant features iteratively based on backward sequential selection by deleting one feature at a time, resulting in a sub-optimal combination of *r *(*r*<*n*) features with best predictive performance.

In this study, the CDSS was designed using SVM embedded with a radial basis function (RBF) kernel. The performance evaluation was conducted using 6-fold cross-validation and the experiments were repeated 10 times for individual cases with different number of samples and different strategies of feature selection. In addition to feature selection method, SVM parameters are also crucial in training a CDSS with good predictive performance. The SVM parameters were determined by using different combinations of regularization parameter (*C*) and kernel parameter (*γ*) with a grid size of 0.1 to select the optimal parameters with best predictive accuracy. As indicated in Table 2, Optimal SVM parameters, i.e. *C *and *γ*, for datasets with different number of accumulated samples in different periods tend to have different optimal combinations of SVM parameters. The CDSS designed with more data samples achieved better predictive performance, that is, higher average accuracy, sensitivity, and specificity.

**Table 2:** Optimal SVM parameters of different datasets containing different number of samples for model construction using different combination of features selected using filter methods (7 features) and wrapper methods (11 features), respectively.

Sample (N)	Feature Selection	Accuracy (SD) (%)	Sensitivity (SD) (%)	Specificity (SD) (%)	log_2_*C*	Log_2_*γ*
348	Filter	88.33 (0.84)	90.32 (1.46)	85.86 (1.18)	32	16

	Wrapper	92.73 (0.79)	95.81 (0.94)	88.97 (1.96)	6.2	3.1

287	Filter	85.19 (1.55)	92.17 (0.87)	73.97 (3.48)	4	64

	Wrapper	90.56 (1.37)	95.14 (2.05)	85.00 (2.34)	5.9	3

231	Filter	78.73 (1.57)	91.08 (1.03)	63.77 (3.28)	0.0625	64

	Wrapper	85.27 (1.57)	92.34 (2.41)	76.35 (2.63)	4.8	3

188	Filter	77.16 (1.16)	86.55 (1.79)	64.91 (1.72)	0.5	8

	Wrapper	79.88 (1.34)	91.42 (1.32)	76.35 (4.13)	6	3.2

According to the plot depicting accuracy against number of features selected based on the SVM-RFE method, a total of 11 selected features achieved the maximum accuracy. [34]. Furthermore, as summarized in Table 2, predictive performance of the filter methods and wrapper methods for selecting salient features and designing SVM classifiers based on the samples collected and accumulated in 4 different periods are compared. It can be observed that the wrapper method (11 features) outperforms the filter method (7 features) in selecting salient variables for the design of SVM classifiers [34]; therefore, the SVM-RFE based on 348 data samples was applied to design the CDSS for predict patients who have greater probability to be weaned from ventilators in clinical setting.

**Figure 4:**
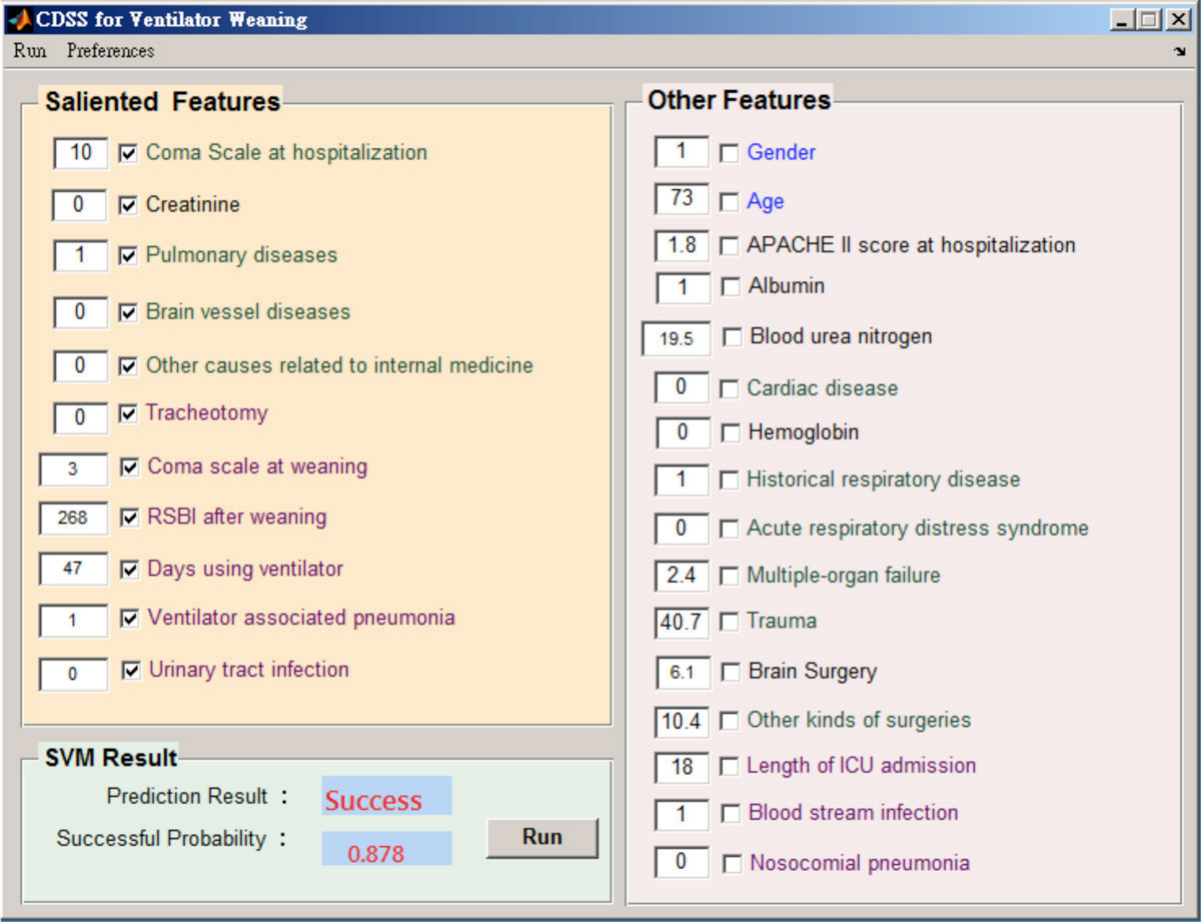
**Graphic user interface of the CDSS for ventilation weaning**.

The graphic user interface (GUI) of the prototypic CDSS is shown in Figure 4. As shown in this figure, the values of 11 salient variables (upper-left corner) can be input and stored for later analysis. If the predicted successful probability is higher than a threshold (0.5), the system suggests the physician to wean the mechanic ventilator from the patient. All the 11 variables had been reported to be related to ventilator weaning. Among them, age is a demographic information; creatinine level, pulmonary disease, brain vessel disease, other causes relate to internal medicine, coma scale at weaning, rapid shallow breath index (RSBI) are physiology and disease factors; and tracheostomy operation, days using ventilator, ventilator associated pneumonia, and urinary tract infection are categorized as care and treatment factors. Some of the medicine-related variables are description as follows:

• *Creatinine *is a chemical molecule produced from muscle metabolism. The kidneys maintain the blood creatinine in a normal range. Creatinine has been found to be a reliable indicator of kidney function. Elevated creatinine level indicates impaired kidney function or kidney disease.

• The Glasgow *Coma Scale *(GCS) is the most common scoring system used to describe the level of consciousness in a patient. Basically, it is used to evaluate the severity of an acute brain injury. Clinicians always use this scale to rate eye opening, verbal, and motor responses that an individual is able to react.

• *Rapid shallow breath index *(RSBI) is a ratio of the patent's respiratory rate (RR) to the value of the minute volume (VE) divided by respiratory rate (RR), that is RSBI = RR/(VE/RR). An RSBI greater than 105 is believed to be associated with a need of intubation and an increase of in-hospital mortality.

• *Tracheotomy *is a hole created by surgery through the front neck and into trachea. A tracheotomy is often needed when a patient with severe health problems requiring long-term use of a ventilator to help breathe.

• *Ventilator associated pneumonia *(VAP) is defined as pneumonia occurring more than 48 hours after patients have been intubated and received mechanical ventilation. Diagnosing VAP requires a high clinical suspicion combined with bedside examination, radiographic examination, and microbiologic analysis of respiratory secretions. VAP is also the most common and fatal infection of ICU patients, highly increasing the risk of mortality as compared with similar patients without VAP.

### Data collection

The following variables were recorded after the patients were admitted: demographic data, APACHE (acute physiology and chronic health evaluation) II Score, GSC (Glasgow coma scale), and blood biochemistry test, including blood urine nitrogen (BUN), Creatinine (Cr), Albumin, and Hemoglobin. Electrocardiogram, blood pressure, heart rate, and SpO_2 _were continuously monitored. The hospital acquired infective diseases were recorded during admission period. Before SBT, the follow variables were recorded: APACHE II Score, GSC, blood biochemistry test (BUN, Cr, Albumin, Hemoglobin), days using mechanical ventilator, ventilatory variable, arterial PaCo_2_, and PaO_2_/FiO_2_. The respiratory variables, including minute ventilation, repertory rate (f) , tidal volume (V_T_), and P0.1 (pressure of 0.1 second after starting expiration), displayed on the ventilator were recorded at the first minute, 30th minute, and 60th minute of the SBT. Measurements were performed by a respiratory technician and were repeated for 3 times separated by an interval no less than 15 second with the obtained mean values being used for data analysis. The RSBI (f/VT) were calculated at the first minute. Endotracheal tube suction was performed 5 minutes before each measurement.

### Statistical analysis

The statistical analysis was performed using the statistical software package (SPSS, version 19.0). The results of descriptive analysis were expressed as mean and standard deviation (SD) for continuous variables and frequency for binary variables. The student's *t*-test and Pearson chi-square test were applied to compare continuous variables and binary variables, respectively, with the level of significance defined as *p*<0.05. The sample size was calculated by G power 3.1 [36], with a two-sided 5% significance level and a power of 80%, a sample size of 50 patients per group was necessary, given an anticipated dropout rate of 10%.

## Results

After excluding 22 and 46 patients in the study group and control group, respectively, data collected from 168 and 144 patients of two individual groups were used for further analyses. The demographic characteristics of the control and study groups are shown in Table 3. As indicated in this table, there is no significant difference between two groups with regard to demographic information, i.e. age and sex; physiological and disease factors, i.e. APACH II score, GCS, Albumin, BUN, creatinine; and care and treatment factors, i.e. tracheostomy, ventilator acquired pneumonia, blood stream infection. This indicates that the subjects in the control and study groups are matched well. After CDSS intervention, it can be found that days using mechanical ventilator for the study group (38.41 ± 3.35) is significantly shorter than the control group (43.69 ± 14.89) with a decrease of 5.2 days in average, reaching a significant level of *p*<0.001 (Student's *t*-test). As shown in Table 4, the sensitivity of CDSS (study group) is 87.7%, which is again significantly higher than the control group (61.4%) with an odd ratio of 4.45 and 95% CI of [1.851,10.815], reaching a level of *p*<0.01 (Pearson χ^2 ^test).

**Table 3:** Demographic and clinical characteristics of two groups.

	Study Group (N = 168)	Control group (N = 144)
Age (years)	77.29 ± 13.34	19.98 ± 13.94

Sex, male (%)	56	59

Apache II score	20.57 ± 4.88	21.4 ± 4.48

Tracheotomy (%)	47.6	45.1

Successful weaning (%)	65.3	52.1

Ventilator acquired pneumonia (%)	17.3	18.1

Blood stream infection (%)	2.4	4.2

Albumin	2.08 ± 0.54	2.11 ± 0.57

BUN	39.64 ± 35.86	41.03 ± 41.58

Creatinine	1.45 ± 1.28	1.50 ± 1.81

GCS	8.48 ± 3.34	8.08 ± 2.91

RSI	145.43 ± 64.15	138.37 ± 63.62

Days using ventilator***	38.41 ± 3.35	43.69 ± 14.89

***Significance with *p*<0.001 using student's *t*-test.

**Table 4:** Comparison of weaning sensitivity between the study and control groups

	Weaning Status		Total
		
	Successful	Failure	
Study Group	57 (87.7%)	8 (12.3%)	65

Control Group	43 (61.4%)	27 (38.6%)	70

Total	100	35	

Note: Significant with a level of p<0.01 (odd ratio= 4.474; 95% CI=[1.851, 10.815]) using Pearson χ2 test.

As shown in Table 5, at the end of the experiment, a total of 178 patients were successfully weaned: 103 (65.3%) in the study group and 75 (52.1%) in control group with an odd ratio of 1.46 and 95% CI of [0.929,2.288]. No significant difference between these two groups was observed (Pearson χ^2 ^test, *p*>0.05), indicating that there was no difference with regard to disease severity between the control and study groups by accounting the maximum duration of 63 days in using the mechanical ventilator.

**Table 5:** Comparison of weaning rate between the study and control groups

	Weaning Status		Total
		
	Successful	Failure	
Study group	103 (65.3%)	65 (38.7%)	168

Control group	75 (52.1%)	69 (47.9%)	144

Total	178	134	

Note: Not significant with a level of p<0.05 (odd ratio= 1.46; 95% CI=[0.929, 2.288]) using Pearson χ2 test.

## Discussions

The American College of Chest Physicians, the Society of Critical Care Medicine, and the American Association for Respiratory Care created five evidence-based guidelines for ventilator weaning based on the following principles: (1) frequent assessment is required to determine whether ventilator support and the artificial airway are still needed; (2) patients who continue to require support should be continually re-evaluated to assure that all factors contributing to ventilator dependence are addressed; (3) with patients who continue to require support, the support strategy should maximize patient comfort and provide muscle unloading by fitting the physical needs for individual patients and tuning the operational modes of ventilators to enable them not to fight the usage of ventilators and to make them feel as comfortable as possible; (4) patients who require prolonged ventilator support beyond the ICU should go to specialized facilities that can provide more gradual support reduction strategies; and (5) ventilator-discontinuation and weaning protocol can be effectively carried out by non-physician clinicians. The designed CDSS is efficient in realizing the first two guidelines under the supervision of physicians. As soon as the mechanical ventilator has been successfully weaned, general healthcare providers will take over the patients.

A number of physiologic indices have been described to predict the outcome of attempts at discontinuing ventilator support. Previous investigations showed that several physiological indexes, such as rapid shallow breathing index (RSBI) [6,14], maximal inspiration pressure (PImax) [15,16], vital capacity (VC) [16], minute ventilation (VE) [15,17], and pH and pCO2 values of stomach mucosa [18], etc., were useful for successfully predicting ventilation weaning. It was also shown that several variables, such as arterial blood gas levels, fraction of inspired oxygen, alveolar-arterial oxygen pressure difference (A-a gradient), blood urine nitrogen (BUN) level, serum creatinine level, serum albumin level, age, and days using ventilator were correlated to successful weaning [19,20]. In addition to BUN level and albumin level [12], race and reason for ventilator dependency were also found to be major predictors. However, some of the above indices are difficult to measure and cannot be applied in daily practice.

Among the aforementioned variables, RSBI [6,14] and days using ventilator [20] were also adopted in this investigation. Specifically, Meade *et al. *[6] found that RSBI is the most frequently studied and one of the most powerful indexes in successful weaning. From our understanding, variables including brain vessel disease, coma scales at weaning, ventilator associated pneumonia, tracheotomy, and urinary tract infection used in this study have never been reported elsewhere as indicators of weaning prediction. More recently, it was reported that variables of mechanical ventilator for different disease states, i.e. healthy lung and acute respiratory distress syndrome (ARDS), were shown to have different target P_a_CO_2 _and respirator rate for automated mechanical ventilation [37], indicating disease factors are crucial variables for predicting successful weaning. Weaning failure is usually multifactor in nature. Although a number of physiologic variables have been described to predict the outcome of attempts at discontinuing ventilator support, variables that assess a single physiologic function are frequently inaccurate predicators. Unfortunately, previous studies only focused on physiological variables. We suggest that other disease and therapeutic progression variables should also be considered.

Chen *et al. *[38] found that patients with lower APACHE II scores had higher incidences of successful weaning from the ventilator in Respiratory Care Center (RCC). In addition, it was reported that long-term survival was inversely associated with age and length of stay in ICU or RCC [20]. However, these three variables were not selected for designing the CDSS. The main reason might be that they are not compatible with other more important selected variables.

In this study we designed and applied the CDSS in clinical setting of a national hospital situated in central Taiwan. As indicated in Table 4, the results show that a sensitivity as high as 87.7% has been achieved in the study group, which is significantly higher (p<0.001) than the weaning determined by physicians only (sensitivity: 61.4%). However, as shown in Table 5, the weaning predictive rates between the control and study groups show no difference (p>0.05), mimicking that the CDSS can make better decision in determining the patients who have greater potential to be weaned than the traditional protocol with weaning determined only by physicians. It was reported that variability of preference toward ventilator settings existed among physicians, resulting in different opinions on the same patients [39], indicating that ventilator weaning determined by physicians tends to have lower sensitivity.

According to the retrospective study [34], the CDSS adopted in this study achieved a predictive accuracy of 91.25%, which outperformed previous studies using f/V_T _as the predictive index achieving accuracies ranging from 75-78% [25,26] and a model using a combination of sample entropy of three variables, i.e. inspiratory tidal volume (VTI), expiratory tidal volume (VTE), and respiration rate (RR), with an accuracy of 78.6% [24]. A predictor designed with variables obtained from a single device tends to incur systematic errors [21]. Hence, the CDSS designed by adopting multiple variables obtained from diverse instruments or modalities demonstrates effective in compensating system errors incurred by variables acquired from a single instrument, which in turn elevates the predictive performance. Furthermore, the results demonstrate that the proposed CDSS is efficient in reducing the period of ventilator use for 5.2 day in average, resulting in a decrease of healthcare cost of NT$45,000 (around US$1500) per patient according to the current National Health Insurance setting of Taiwan.

Over the past few decades many CDSS have been developed for mechanical ventilation setting, but few have been applied in the clinical setting for weaning patients from mechanical ventilation. This study demonstrates the ability of the CDSS to safely and effectively wean patients with complex medical problems. The limitation of this study is that only the sensitivities of the two groups were compared, since, in the prospective study, it is impossible to obtain the specificity of weaning prediction.

## Conclusion

The clinical decision support system (CDSS), designed based on demographic information, physiology and disease factors, and care and treatment factors, has been demonstrated to be effective in the identification of the earliest time of ventilator weaning for a patient to resume and sustain spontaneous breathing, thereby avoiding unnecessary prolongation of ventilator use and decreasing healthcare cost. By identifying patients who are likely to fail a trial of spontaneous breathing, such variables can prevent a premature weaning attempt and the development of severe cardiorespiratory and/or physiological decompensation.

## Competing interests

The authors declare that they have no competing interests.

## Authors' contributions

JCH conducted patient recruitment and clinical test; JCH and YFC designed the experimental paradigm and wrote the paper; WSC, THT, and YFC contributed to the discussion of the work; TC and JYC proofread the manuscript. All authors read and approved the final manuscript.
